# Human Expressions of Object Preference Affect Dogs’ Perceptual Focus, but Not Their Action Choices

**DOI:** 10.3389/fpsyg.2020.588916

**Published:** 2020-11-06

**Authors:** Enikő Kubinyi, Flóra Szánthó, Elodie Gilmert, Ivaylo B. Iotchev, Ádám Miklósi

**Affiliations:** ^1^Department of Ethology, ELTE Eötvös Loránd University, Budapest, Hungary; ^2^Laboratoire d'Ethologie Expérimentale et Comparée, Université Paris 13, Villetaneuse, France; ^3^MTA-ELTE Comparative Ethological Research Group, Budapest, Hungary

**Keywords:** emotion recognition, desire state attribution, object choice test, disgust, dog

## Abstract

Inspired by work on infants, we investigated whether dogs’ behaviors are guided by human displays of preference, contrasting with the animals’ own choices. In a rewarded fetching task, dogs override their own interest toward “disgusting” objects and retrieve what the owner prefers. However, in previous research, both objects were inherently neutral to the dogs and they might have chosen the owner’s object because a “happy owner” predicts a positive outcome. If dogs are indeed able to override their own interests, we expected them to fetch the owner’s object even if (1) they would prefer another one and (2) do not receive a reward for it. Two objects were compared, a toy (hoop) and a bracelet. After establishing that the toy was preferred by all dogs in an initial test of preference, we applied a two-choice procedure to test if either fetching or looking at the objects from a distance would be affected by the owner’s choice. In Study 1, the owner demonstrated happiness toward the bracelet and disgust toward the toy with both facial and body gestures accompanied by verbalizations. Then the owner asked the dog to fetch, without providing additional guiding cues. All dogs fetched the toy, indicating that their own choice was not overcome by the positive emotional state signaled by the owner. To avoid direct contact with the objects, in Study 2 we placed the objects on an unreachable spot after the emotion demonstration and measured the duration of looking at the objects. In the “bracelet” (non-matching) group the owners demonstrated happiness toward the bracelet and disgust toward the toy, similar to Study 1. In the “toy” (matching) group the owners showed happiness toward the toy and disgust toward the bracelet. When the objects were placed on the unreachable spot, dogs looked at both objects for the same amount of time in the non-matching group, but longer at the toy in the matching group. Although the studies did not demonstrate that dogs override their own preferences for an object, the results suggested that the owners’ expressed preference was perceived by the dogs and guided their perceptual focus.

## Introduction

Studies on the cognitive and emotional development of pre-verbal children often face similar challenges as those conducted with non-human animals. Inspired by work on infants (Repacholi and Gopnik, 1997) we decided to test dogs’ ability to use human emotional expressions as an informative cue in a two-choice task in which the dogs’ own preferences for the options competed with the expressions humans made toward the choices. Repacholi and Gopnik (1997) investigated whether human infants understand subjectivity of the desire, i.e., that different people can have different attitudes toward the same object. They used two types of food (cracker and broccoli) and created two groups. These groups differed in terms of which food the experimenter desired, with the underlying assumption that participants would exhibit a strong preference for one food (cracker). The results indicated that 18-month-old children offered the food to the experimenter, which she previously preferred, even in cases when the children did not prefer this particular food (broccoli). In contrast, 14-month-old children offered the crackers (i.e., the food they preferred), regardless of the experimenter’s preference display. Based on the results, the authors suggested that 18-month-olds can infer other’s preferences and they recognize how desire can be inferred from emotional expressions.

We hypothesized that similar to infants, companion dogs’ behavior might also be influenced by expressions signaling the owner’s desire, even if it is in contrast to their own. Several studies provided evidence that dogs are able to discriminate between human facial expressions. Nagasawa et al. (2011) tested the ability of dogs to discriminate blank from smiling faces of their owner in a two-object choice task and whether the sensory learning would generalize to novel pictures, including those of unfamiliar people. Dogs were also shown to be sensitive to ostensive cues (Tauzin et al., 2015) or, in case of the old cohort, human voices with different valence (Smit et al., 2019). In two-object choice tasks, dogs chose objects which were attended to humans with facial expressions signaling preference (Prato-Previde et al., 2008; Buttelmann and Tomasello, 2013; Merola et al., 2014; Turcsán et al., 2015). However, as these studies used neutral stimuli for the dogs (e.g., identical plastic bottles for both the positive and the negative situation in Turcsán et al., 2015), it is unclear whether dogs are able to differentiate between their own preference and that of the owner. A study by Prato-Previde et al. (2008) used a contrast between the owner’s preference expressed for two quantities of food and the dogs’ inherent preference for the larger amount, but the effect of the owners’ expressed preference was strongest when quantity information was removed by offering two equally small amounts.

Importantly, information about others’ internal states (e.g., preference) might be utilized differently between species, leading to differences in which behaviors are affected. In comparisons between human children and young chimpanzees (Warneken, 2006) behaviors indicative of altruistic motives or prosocial helping are more strongly expressed in human children. Although dog social cognition appears to have adapted to the human social environment (Hare and Tomasello, 2005; Topál et al., 2009) there is also evidence accumulating that dogs can be more competitive and/or less prosocial compared to their closest wild relative – the wolf (Range and Virányi, 2014; Dale et al., 2017, 2019, 2020).

Not only prosociality but also inhibitory control (the ability to overcome an immediately rewarding behavior in favor of a delayed and ultimately more rewarding one) affects social decision making, i.e., the extent of how goal-directed behaviors are affected by others’ preference (Macphail, 1970; Hulbert and Anderson, 2008; Bari and Robbins, 2013). In other words, actively helpful behavior might require the suppression of one’s own preference in addition to being able to perceive what others want. In children, self-control can inhibit the impulse to act selfishly as altruistic 4–6-year-old children perform better on an inhibition task than non-altruistic children do (Aguilar-Pardo et al., 2013). Dogs also vary in impulse control demonstrated in a touch screen test (Bunford et al., 2019), but a link between inhibitory control and social behavior was found so far only for the expression of inequity aversion, which is stronger in dogs with higher inhibitory control (Brucks et al., 2017), but not for cooperative behaviors (Dale et al., 2020). The capacity to inhibit prepotent responses can vary significantly even between taxa (linked to the maturity of the dorsolateral prefrontal cortex), e.g., capuchins (*Cebus apella*) easily inhibit the tendency to reach directly for food but tamarins (*Saguinus oedipus*) do not, despite extensive training (Lakshminarayanan and Santos, 2009). Importantly, we expect inhibition to play a role in how animals react to communicative signals, even if emotional expressions are not received as information about internal states. Expected rewards associated with a satisfied human may compete with rewards deriving from own preferences, in which case being able to choose the former over the latter may also require inhibitory control.

In light of this literature, we consider here that sensitivity for others’ internal states, like preference, might not necessarily show up in active behaviors. Dogs may lack the self-control to overcome contact with their preferred object and therefore we will test not only fetching but also looking orientation when the objects are unreachable. We assume that the owner’s preference expression might causally impact dogs’ behavior. The latter, looking duration and orientation, has been shown on several occasions to reflect a relocation of attention/interest in dogs (Miklósi et al., 2003; Bognár et al., 2018; Petrazzini et al., 2020) and possibly also to signal communicative intent (Miklósi et al., 2000). Given that actively helpful behaviors, like offering (Repacholi and Gopnik, 1997), might be strongest in human children (Warneken, 2006) we expect that the perceived preference of others might be less influential on behaviors like fetching, and more visible in measures of looking time as an operationalization of perceptual focus in dogs. The influence of human preferential expressions is also expected weaker in the fetching condition if we assume that the underlying driving force of dogs’ behavior is a competition between social and non-social rewards. Objects in reach qualify as strong affordances (Gibson, 1977) and might distract from socially cued rewards.

The present study is a direct follow-up of research Turcsán et al. (2015), where the authors claimed that “dogs are able to recognize which is the more positive among two emotions, and in a fetching task situation, they override their own interest in the ‘disgusting’ object and retrieve what the owner prefers.” However, overriding their interest was “easy” for the dogs in the cited study, as the two objects (plastic bottles) were originally neutral for the dogs and they could simply choose the positively marked object upon request because a “happy owner” predicts a positive outcome. In order to investigate whether dogs indeed link the owner’s emotional expression with his/her internal state and not with the associated reward, the valence difference between objects in the two-choice paradigm has to be different from each perspective: one should be more attractive for the owner, and the other more attractive for the dog. We expect that if dogs are indeed able to override their own interest, they will fetch the owner’s object even if they would prefer another one and they do not receive a reward for it. Therefore, (1) we used two objects with different inherent valence, one clearly preferred by the dogs and (2) we have not rewarded the dogs for their choice, contrary to previous studies when the choice of objects marked by the positive emotional expression of the owner resulted in food (Buttelmann and Tomasello, 2013; Turcsán et al., 2015) or toy rewards (Merola et al., 2014), which may have affected the choice behaviors of the subjects.

Although in the study of Repacholi and Gopnik (1997) it was the experimenter who demonstrated emotions, we asked owners to fulfill this task. In spite of possible limitations (owners are not professional actors), previous work has shown that dogs distinguish better between positive and negative emotional expression of their own owners compared with an unfamiliar experimenter (Merola et al., 2014).

To sum up, our main goal was to investigate whether dogs’ behaviors are guided by human displays of preference, contrasting with the animals’ own choices. How information about preference is exactly transmitted and what it means to the receiver is outside the scope of the present work, however. Note, that in externally observable behaviors, mechanisms like empathy or communication cannot always be distinguished (Miklósi, 2009).

## General Methods

### Ethical Statement

The behavioral observations conducted in this study complied with national (Hungarian law: “1998. évi XXVIII. Törvény” 3.§/9. – The Animal Protection Act) and EU legislation, as well as institutional guidelines. The Hungarian “Animal Experiments Scientific and Ethical Committee” approved the experimental procedures under the numbers: PE/EA/2019-5/2017. Owners provided written consent to their participation. Our Consent Form was based on the Ethical Codex of the Hungarian Psychologists (2004). We took special care to ensure that the consent process was understood completely by the participant. In the Consent Form participants are informed about the identity of the researchers, the aim, procedure, location, expected time commitment of the experiment, the handling of personal and research data, and data reuse. The information included the participant’s right to withdraw their consent at any time. Participants could easily (and without penalty) decline to participate and could ask not to use or delete data collected during the experiments.

### Location and Setup

**Figure 1:**
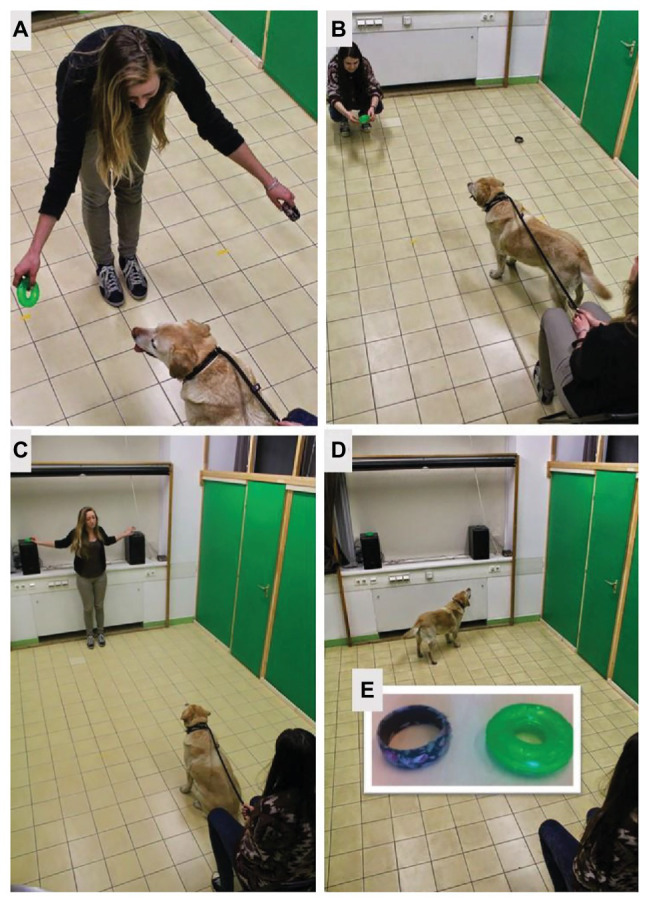
**(A)** Object preference test, **(B)** demonstration by the owner, **(C)** object hiding in Study 2, **(D)** looking at the objects in Study 2, **(E)** test objects for all dogs: bracelet (left), toy (right), both 9 cm in diameter. The persons identifiable in the images provided written consent for the publication.

The tests took place in a 5 × 2.5 m room. Only the dog, the owner, the experimenter, and a chair were present in the room. There were markings on the floor indicating the locations of the objects (1.5 m apart from each other and 2.5 m apart from the subjects’ starting place) and also a chair for the owner (Figures 1A–D).

The experimental objects were two objects, a toy, and a bracelet. The toy was a green, flexible rubber ring (a hoop), 9 cm in diameter, the bracelet was a black plastic ring with purple flower-patterned textile cover, 7.5 cm in diameter (Figure 1E). The objects were cleaned after each test.

The tests were video-recorded from the time-point when the dog entered until it left the room and used later for behavior coding. Dogs were free to explore the room for 5–6 min before the trials, while the experimenter was instructing the owner. After a few minutes, the dogs were standing/sitting/lying passively, suggesting that the habituation period was long enough to decrease potential stress due to the new situation.

Dogs were recruited on a voluntary basis from the Family Dog Project database in Budapest, Hungary. Only those dogs that knew the “fetch” command (in Hungarian) according to their owners were included.

## Study 1: Fetching Objects

We investigated whether dogs make choices based on the owners’ preference, and therefore fetch the object, which is associated with their owner’s positive emotional expression, despite their own preference to the contrary.

### Subjects

Twelve dogs (eight mixed-breeds, border collie, golden retriever, Labrador retriever, dachshund, mean age +/− SD = 3.80 +/− 1.17 years, age range: 1.5–8.5 years, five males, seven females) were studied.

### Object Preference Test

We observed which object was preferred by the dog. The owner sat down on the chair and held the dog on a leash. In front of them, the experimenter showed the two objects to the dogs (i.e., put both objects in front of the dogs’ nose) for 3–4 s. One object was held in the right hand, the other in the left (randomly). After the dog smelled both objects, the experimenter opened her arms (Figure 1A). The dog was free to move toward the objects. If the dog tried to grab the object, the experimenter took it away and after a few seconds, she opened her arms holding the objects again. We observed which object was followed by the dog. If the choice was unclear because the dog has not followed either object, the trial was repeated. After a clear choice (i.e., the dog oriented toward/touched one object continuously for at least 5 s), the experimenter gave the objects to the owner, took the leash of the dog, and instructed the owner about the setup of the following demonstration phase (starting side and the order of the emotions).

### Demonstration by the Owner

After the object preference test, the owner stood up, showed both objects in front of the dog, then backed 3 m, and put down the objects 2 m away from each other. Then s/he crouched down behind one object, touched it, looked at the dog, and gave the instructed emotional expression (happy for the bracelet and disgust for the toy) for 3–4 s (Figure 1B). Concerning the demonstration, we followed the protocol of Turcsán et al. (2015). The owners displayed both facial and body gestures accompanied by verbalizations. The owners were instructed that they should try to display these emotions as they usually do, e.g., when they try to invite the dog to play or when their dog found something particularly distasteful. They were not allowed to use any word known as a command for the dog during the demonstration.

**Figure 2:**
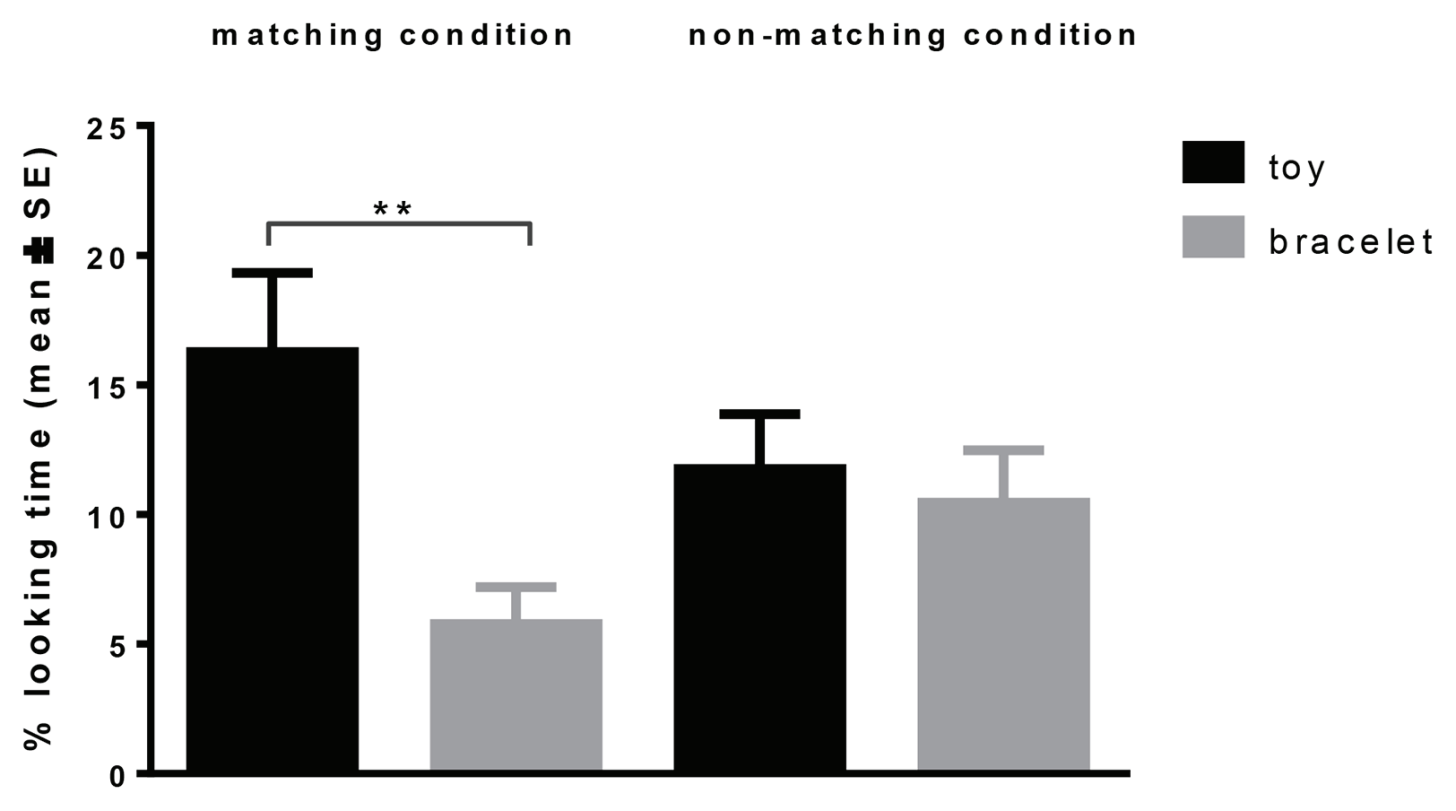
Duration of looking time (in percent) at the toy and the bracelet in the matching (owner reacts happy toward toy, with disgust toward bracelet) and non-matching (owner reacts happy toward bracelet, with disgust toward toy) condition in Study 2. Significant differences are indicated (^**^p < 0.01).

Then the owner put the object back in its place, walked to the other object, and repeated this display with the other assigned emotion. During the demonstration, the experimenter stood silently behind the dog, looking toward a point halfway between the objects. After the demonstration, the owner left the objects on the floor, walked back to the chair, sat down, grabbed the leash, and positioned the dog in the middle, facing toward the objects.

### Fetching

The owner released the dog and immediately gave the “Hozd” verbal command (“Fetch” in Hungarian). The owner was strictly instructed not to use any gestures or directional cues and they were required to look straight ahead between the objects while giving the command. If the dog started to move toward the objects, the owner stopped talking and sat silently and motionless. After the dog fetched one of the objects to the owner, the dog was briefly praised by the owner. During this phase the experimenter stood silently next to the owner, looking at a point halfway between the objects. The maximum duration of the fetching phase was 1 min. Next, the experimenter retrieved both objects, and the next trial started with the Demonstration phase.

Each dog received four trials, the side of the objects and the direction of the demonstration (from left to right or vice versa) changed randomly in every trial. We recorded whether the dog fetched the toy or the bracelet during the fetching of an object phase.

### Statistical Analysis

We used only descriptive statistics in Study 1. The behavior of eight dogs were coded by a second observer. The two observers agreed fully regarding both the object preference and the fetched object variable.

### Results and Short Discussion

In the object preference tests, all dogs chose the toy. In the fetching test all dogs fetched an object at least in one trial: one dog in one trial, four dogs in three trials, and seven dogs in all four trials. From the altogether 41 fetching events, the bracelet, that was preferred by the owner during the demonstration phase, was fetched only 2 times (5%) by two dogs (one dog fetched the bracelet in the second, the other in the fourth trial). Therefore, dogs fetched the object which was preferred by them (the toy), and not the object that which was preferred by the owner. Thus dogs either (1) are not able to distinguish between their and the owners’ preference based on his/her happy emotions toward the bracelet or (2) their own preference has not been overwritten by the positive emotional state signaled by the owner (i.e., they did not inhibit the “wrong” response; Bari and Robbins, 2013). The second interpretation can alternatively concern a competition between rewards associated with the toy and with a happy owner, but in both variants we assume that acting upon the toy was not suppressed in favor of acting upon the owner’s social referencing. Moreover, we assumed that an object in reach acts as a stronger affordance (see Gibson, 1977), while previous work also had shown that objects out of reach stimulate what appears to be “showing” behavior in dogs (Miklósi et al., 2000) suggesting that placing the objects out of reach could stimulate dogs to direct more attention toward their owners, thereby also weakening the affordance provided by their preferred object. To test the second hypothesis, we decided to put the objects at unreachable positions, thereby preventing direct contact, which likely decreases the play drive. We assumed that the time spent looking at the toy or bracelet would reflect the owner’s expressed preference. In particular, we expected the longest looking duration for the object preferred by both dog and owner and the shortest for the object disliked by both. Thus, a significant difference between time spent looking at the toy and bracelet was expected in the matching condition (owner expressing a preference for the toy).

## Study 2: Looking at the Objects

In this study, both objects were placed out of reach when the owner was asking for them and this way we could compare looking time and orientation between two groups of dogs: one with owners who preferred the same object as the dog (the toy), and one with owners who preferred the other object than the dog (the bracelet). We expected the greatest difference in looking times between the two objects in the matching condition (preference expressed for the toy, but disgust toward the bracelet), while in the non-matching condition looking times should differ less as a result of interference between own and other’s perceived/inferred preference.

### Subjects

Fifty one dogs, naïve to the procedure of the previous study, were assigned to two groups.

In the matching condition (Toy group) 26 dogs participated (1–10 years old, mean age = 3.55, SD = 2.23 years, 50% males, 58% neutered, eight mixed breed dogs, three golden retrievers, two English bullterriers, two Staffordshire bullterriers, two beagles, Labrador retriever, English cocker spaniel, whippet, miniature schnauzer, great Dane, pumi, Cavalier King Charles spaniel, labradoodle, border collie).

In the non-matching condition (Bracelet group) 25 dogs were involved (1.3–8.5 years old, mean age = 3.89, SD = 2.14, 44% males, 72% neutered, 13 mixed breed dogs, two golden retrievers, three Labrador retrievers, border collie, miniature dachshund, Airdale terrier, bichon Havanese, standard poodle, Transylvanian hound, beagle).

### Object Preference Test

The test was the same as in Study 1.

### Demonstration by the Owner

In the matching condition (toy group), the owner displayed happiness toward the toy and disgust for the bracelet. In the non-matching condition (bracelet group), s/he displayed happiness toward the bracelet and disgust for the toy. Otherwise the procedure was the same as described in Study 1.

### Object Hiding

After the demonstration, the owner went back to the dog, sat down on the chair, and held the dog on a leash. The experimenter went to the objects, put both objects on the window sill 2 m apart from each other (Figure 1C), out of reach from the dog, and went back next to the chair.

### Object Requesting

The owner let the dogs free and said “Hozd” to the dog (“Fetch” in Hungarian). Owner was instructed not to use any directional cues and look directly ahead. The dog was free to move in the room and could see the objects but could not reach them. The length of the phase was 30 s (Figure 1D). Duration of “looking at the bracelet” and “looking at the toy” behavioral variables were measured (as %, by dividing them with the total time of this phase).

### Statistical Analysis

The variables were coded by a second observer for eight subjects. We evaluated the inter-observer reliability using two-way random intraclass correlation (ICC, McGraw and Wong, 1996), looking for absolute agreement between average measures. ICC was 0.706 for looking at the bracelet and 0.764 for the looking at the toy variable. During the looking at the object test three dogs did not look at any objects (one dog from the toy, two dogs from the bracelet group); these dogs were excluded from further analysis. four dogs (two from both groups) had to be excluded because of technical issues (problems with following the protocol). Therefore, the final sample sizes consisted of 23 dogs in the toy and 21 dogs in the bracelet group.

A generalized linear mixed model (GLMM) was used to investigate how looking duration differed between objects and conditions. In particular, the initial model included the predictors age, sex, reproductive status, condition (2 levels: matching vs. non-matching) and object (2 levels: toy vs. bracelet), as well as the interactions sex × reproductive status and condition × object. A model with a Gamma distribution assumption (Nelder and Wedderburn, 1972), a robust test of coefficients and a Satterthwaite method for calculating the degrees of freedom was specified, since the assumption of normal distribution was violated (Kolmogorov-Smirnov test of normality for the residuals of looking duration, *p* < 0.001). The model was optimized by backwards elimination combined with an Akaike information criterion, i.e., the least significant predictors that were not part of an interaction were removed until an optimal (smallest) Akaike value was reached. Prior (control) and post-hoc analyses consisted of *t*-tests (paired *t*-tests for within condition comparisons and independent samples *t*-tests for between condition comparisons). All analyses were conducted in SPSS v25.

### Results and Discussion

Condition had no effect on the total proportion of time spent looking at either object (independent samples *t* = −0.037, *p* = 0.971), i.e., on average dogs in each condition spent 22% of the time looking at any object (either toy or bracelet).

The final model predicting looking duration (% of total trial time) included the factors condition, object, and their interaction. The interaction condition × object was significant (GLMM, F_1,80_ = 4.585, *p* = 0.035). Dogs looked longer at the toy than the bracelet in the matching condition (16.3 ± 3 vs. 6.3 ± 1.5, % looking duration, means ± SE; *t*_80_ = 2.986, *p* = 0.004, Figure 2), but not in the non-matching condition (*p* = 0.636). Between conditions, there was a trend for longer looking times directed at the bracelet in the non-matching condition (11 ± 2 vs. 6.3 ± 1.5, % looking duration, means ± SE; *t*_80_ = 1.878, *p* = 0.064). No difference was found between conditions for looking duration toward the toy (*p* = 0.285).

The demonstration of the owners affected the dogs’ behavior. If the preference of the owner and the dog matched, dogs looked more at the preferred object (the toy) than the non-preferred (bracelet). If the preference did not match between the dog and the owner, the time of looking at the object which was preferred by the owner, but not the dog (i.e., the bracelet), showed a trend to increase. The result suggests that dogs’ looking behavior is influenced by an interaction between the preference of the owner and their own preference. In particular, the pattern observed between conditions implies that dogs’ own preference and aversion were amplified if matching with the owner’s demonstration since a difference in time spent looking at each was significant only in the matching condition.

It is not certain from these results, however, if the emotional expression or the orientation of the owner’s gaze were the relevant key stimulus. Therefore, in a further study, we investigated whether the owner’s gaze during the object requesting phase is indicative for the dogs during the object choice phase.

## Study 3: The Effect of Directed Gaze

With this study, we investigated the “Clever Hans effect,” i.e., whether owners guide their dogs with minor clues, unnoticeable to the human observer. Therefore. here, we tested whether dogs follow a major clue, i.e., directed gaze. If not, most probably they do not follow minor clues either.

We asked owners during object requesting to directly look either to the object positioned at the right or the left (identical pots were placed in both locations) and investigated whether the dogs’ looking behavior is linked to the gazing direction of the owner.

### Subjects

Eleven dogs, naïve to the procedures of the previous studies, participated in this test (mean age = 4.95, SD = 3.15 years, 54.5% males, 27.3% neutered, three mixed breeds, two German shepherd dogs, 1-1 Labrador retriever, Parson Russel terrier, Yorkshire terrier, sheltie, whippet, Pembroke welsh corgie).

### Training

The dogs were trained to search and fetch a dog toy from a brown, non-transparent flower pot. The owner sat on the chair and asked the dog to sit in front of him/her, facing toward the experimenter who stood in front of them. The experimenter put a pot in front of the dog on the floor, showed the toy to the dog and then put it into the pot. The owner asked to fetch the toy then she gave the toy back to the experimenter. The training trial was repeated twice, with the pot positioned 1 meter from the starting position on trial 1 and 2 m on trial 2.

### Object Hiding

The experimenter asked the owner to put the dog on the leash and sit on the chair. Then she put the toy in her pocket with her back to the dog so the dog could not witness this procedure. Then she put two identical, empty pots on the window sill 2 m apart from each other, out of reach of the dog, and went back to the starting place, similarly to Study 2.

### Object Requesting

This phase was similar to Study 2 save for the owner was instructed to look at one of the pots during the test phase; the direction was balanced between the owners.

### Results and Discussion

In the object requesting test, dogs did not look significantly more or less at the pot which was being watched by the owner (pot watched by the owner: 11.67 ± 2.63 vs. pot did not watch by the owner: 17.23 ± 4.05%, means ± SE; paired *t* = 1.039, *p* = 0.323). The looking direction of the owners during the test did not influence the dogs’ choice.

## General Discussion

Our study aimed to investigate how dogs choose between two different objects if one (a toy) was more attractive to them, but their owner displayed preference for another object (a bracelet). In Study 1, we found that dogs did not fetch the object, which was more attractive for the owner more often. However, when the objects were at an unreachable position in Study 2, dogs’ looking orientation was aligned more strongly with their own preference if the owners’ expressed preference was matching. The interaction between condition and object in Study 2, as well as a trend for increased looking toward the less preferred bracelet in the non-matching condition, suggest that looking times, but not fetching, were influenced by the owner’s expression of preference, but not with his/her potential directional gaze during the object request phase.

It is not certain that this influence is the result of inferred and shared representations (as in Meltzoff’s “Like Me” hypothesis; Meltzoff, 2005), since in theory, human emotional expressions could also act as sign stimuli that induce attentional modulation directly, without intermediate cognitive processing. The emotional cues may, for example, act as local enhancers to guide the dogs’ attention (Arbilly and Laland, 2014). The fact that preference demonstration (by the owner) and measures of looking responses were not simultaneous, argues against the objects being enhanced in a way similar to what is seen in local enhancement, however. Indeed, social referencing has been associated before with effects lasting beyond immediate demonstration (Fugazza et al., 2018) and thus the underlying process must be regarded as more complex. Another reason to exclude simple stimulus enhancement is that an expression was demonstrated toward both objects, thus the underlying mechanism is sensitive to the valence of the referential expression. Considering that dogs seem better at distinguishing strongly opposing emotional expressions from each other than emotional vs. neutral expressions (Nagasawa et al., 2011), it is crucial that in Study 3 neutral gazing alone did not influence the looking direction of the animals.

Comparison with the study by Repacholi and Gopnik (1997), conducted in 14- and 18-month-old children, is somewhat limited since the children’s understanding of others’ desires was operationalized by their offering behavior. It can be assumed, as proposed here, that fetching is a functional equivalent in dogs, but this relies on further assumptions about the underlying cognition of the behavior (e.g., that dogs understand fetching as an act of offering an object to a human). Since only looking behavior was influenced here by the owner’s preference in Study 2, it is possible that response inhibition, an important aspect of cognitive control (Macphail, 1970; Hulbert and Anderson, 2008; Bari and Robbins, 2013), was not sufficiently strong to overwrite the animals’ own preference in Study 1. Dogs’ ability to inhibit their behavior is considered a hallmark of domestication (Hare and Tomasello, 2005; Hare et al., 2012), but differences to wolves regarding this capacity vary based on the type of task (Marshall-Pescini et al., 2015; Brucks et al., 2019) and exhibit a wide variation between individual animals (Brucks et al., 2017). Its relationship to social cognition and behavior is also not uniform and appears more relevant for the expression of inequity aversion (Brucks et al., 2017) than cooperation (Dale et al., 2020). Weaker inhibition as a possible explanation will need to be demonstrated more directly in the future. Interestingly, freeing the owner from a closed space is an active behavior more likely (than fetching) to align with the owner’s expressed emotion (Carballo et al., 2020; Van Bourg et al., 2020). Our results suggest that in the above studies, the dogs’ interest to remain close to the owner (Topál et al., 1998) and the owner’s display of distress might enhance one another, since we also observed a stronger alignment between dogs’ preference and looking orientation, if it was matching with the owner’s expressions.

Other reasons that dogs’ fetching behavior in this study does not match with the offering behaviors of infants (Repacholi and Gopnik, 1997) might relate to uniquely human aspects of early prosocial development. Human infants display signs of altruistic sharing and fairness concern surprisingly early (as young as 15 months; Schmidt and Sommerville, 2011). Although the extent and limitations of early human altruism are still debated (Wynn et al., 2018), it seems stronger in human children than in young chimpanzees (Warneken and Tomasello, 2009). It is thus possible that some forms of responding to others’ preferences are uniquely human. Some work additionally suggests that dogs are more competitive and less prosocial than wolves (Range and Virányi, 2014; Dale et al., 2017, 2019), which might interact with how potential capacities to be influenced by the internal states of others are expressed in measurable behavior. The latter has been demonstrated for imitation (Range and Virányi, 2014), which is less accurate in dogs compared to wolves.

Yet another limitation with using fetching to operationalize sensitivity for owner’s preferences concerns the embodied nature of self-other representations, discussed with regard to imitation for children (Kee, 2020) and also dogs (Topál et al., 2006). Within this framework, it is crucial that fetching is not part of a shared motor repertoire (between humans and dogs) and hence the behavior by itself might prime a more egocentric response.

Finally, a completely non-social explanation can be applied to how dogs’ responded in the fetching task (compared to the looking task). This approach is compatible with the already suggested role of inhibitory control (Macphail, 1970; Hulbert and Anderson, 2008; Bari and Robbins, 2013), but makes no assumption about (shared) internal states. In this scenario the reward from obtaining a preferred object is competing with the expected reward of a satisfied owner. Objects of preference within reach might more likely present affordances (Gibson, 1977), whereas out of reach objects signal the need to attend to potentially helpful humans (see for example, Miklósi et al., 2000). To disentangle this interpretation from hypotheses relying on social cognition and (shared) representations should guide future efforts in the same direction. One important aspect to study in the dog is whether, as in human children (Doan et al., 2015), observing two other agents expressing conflicting preferences, can affect how the animals respond to mismatch involving their own preference. Other factors to control for in the future, concern the duration of dog ownership, which was shown to affect how sensitive the animals were to their owners expressed emotions (Katayama et al., 2019).

Overall, we can conclude that the preference of the owner influenced the dogs’ looking orientation, aligning with previously reported instances of social referencing (Prato-Previde et al., 2008; Turcsán et al., 2015; Fugazza et al., 2018).

The novelty of the results relates to the use of contrasting preferences between the observer (dog) and observed (owner). Although in the study of Turcsán et al. (2015) dogs’ fetching behavior was influenced by their owner’s preference, the dog’s own preference did not play a role (as identical objects were used and the owner’s preference was the only difference). Pongrácz et al. (2013) showed that a dog’s choice of hidden food to be influenced by the owner, but the animals’ knowledge of the preferred food’s position might have played a role, as performance tilted toward the dogs’ preference in later trials. Moreover, in that study the cues were not emotional expressions of preference, but distal pointing cues. While Prato-Previde et al. (2008) used expressions of preference to influence how dogs choose between quantities of food, the expressed preference of the human informants was competing with dogs’ certainty of their own quantity judgments rather than their preferences. The present work, therefore, is the first to our knowledge to directly address how conflicting preferences of self and other influence the behavior of dogs and therefore deepens our understanding of the perception, social cognition, and sensitivity to emotional expressions in these animals. Future studies will need to address, however, if competing social and non-social expected rewards might present a potential alternative explanation for social interpretations of the observed behaviors.

## Data Availability Statement

The raw data supporting the conclusions of this article will be made available by the authors, without undue reservation.

## Ethics Statement

The animal study was reviewed and approved by Hungarian “Animal Experiments Scientific and Ethical Committee, PE/EA/2019-5/2017.” Written informed consent was obtained from the individual for the publication of any potentially identifiable images or data included in this article.

## Author Contributions

EK, FS, and ÁM: conceptualization. EK and FS: methodology. EK and II: formal analysis and visualization. EK and ÁM: resources. FS and EG: data curation. EK, FS, and EG: writing-original draft preparation. EK: supervision and funding acquisition. SF and EG: project administration. All authors contributed to the article and approved the submitted version.

### Conflict of Interest

The authors declare that the research was conducted in the absence of any commercial or financial relationships that could be construed as a potential conflict of interest.

## References

[ref1] Aguilar-Pardo D. Martínez-Arias R. Colmenares F. (2013). The role of inhibition in young children’s altruistic behaviour. Cogn. Process. 14, 301–307. 10.1007/s10339-013-0552-6, PMID: 23436211

[ref2] Arbilly M. Laland K. N. (2014). The local enhancement conundrum: in search of the adaptive value of a social learning mechanism. Theor. Popul. Biol. 91, 50–57. 10.1016/j.tpb.2013.09.006, PMID: 24044984

[ref3] Bari A. Robbins T. W. (2013). Inhibition and impulsivity: behavioral and neural basis of response control. Prog. Neurobiol. 108, 44–79. 10.1016/j.pneurobio.2013.06.005, PMID: 23856628

[ref4] Bognár Z. Iotchev I. B. Kubinyi E. (2018). Sex, skull length, breed, and age predict how dogs look at faces of humans and conspecifics. Anim. Cogn. 21, 447–456. 10.1007/s10071-018-1180-4, PMID: 29667089 PMC6112419

[ref5] Brucks D. Marshall-Pescini S. Range F. (2019). Dogs and wolves do not differ in their inhibitory control abilities in a non-social test battery. Anim. Cogn. 22, 1–15. 10.1007/s10071-018-1216-9, PMID: 30284077 PMC6326967

[ref6] Brucks D. Range F. Marshall-Pescini S. (2017). Dogs’ reaction to inequity is affected by inhibitory control. Sci. Rep. 7:15802. 10.1038/s41598-017-16087-w, PMID: 29150666 PMC5694007

[ref7] Bunford N. Csibra B. Peták C. Ferdinandy B. Miklósi Á. Gácsi M. (2019). Associations among behavioral inhibition and owner-rated attention, hyperactivity/impulsivity, and personality in the domestic dog (*Canis familiaris*). J. Comp. Psychol. 133, 233–243. 10.1037/com0000151, PMID: 30394783

[ref8] Buttelmann D. Tomasello M. (2013). Can domestic dogs (*Canis familiaris*) use referential emotional expressions to locate hidden food? Anim. Cogn. 16, 137–145. 10.1007/s10071-012-0560-4, PMID: 22960805

[ref9] Carballo F. Dzik V. Freidin E. Damián J. P. Casanave E. B. Bentosela M. (2020). Do dogs rescue their owners from a stressful situation? A behavioral and physiological assessment. Anim. Cogn. 23, 389–403. 10.1007/s10071-019-01343-5, PMID: 31907679

[ref10] Dale R. Marshall-Pescini S. Range F. (2020). What matters for cooperation? The importance of social relationship over cognition. Sci. Rep. 10:11778. 10.1038/s41598-020-68734-4, PMID: 32678194 PMC7366628

[ref11] Dale R. Palma-Jacinto S. Marshall-Pescini S. Range F. (2019). Wolves, but not dogs, are prosocial in a touch screen task. PLoS One 14:e0215444. 10.1371/journal.pone.0215444, PMID: 31042740 PMC6493736

[ref12] Dale R. Range F. Stott L. Kotrschal K. Marshall-Pescini S. (2017). The influence of social relationship on food tolerance in wolves and dogs. Behav. Ecol. Sociobiol. 71:107. 10.1007/s00265-017-2339-8, PMID: 28725102 PMC5493712

[ref13] Doan T. Denison S. Lucas C. G. Gopnik A. (2015). “Learning to reason about desires: An infant training study” in *Proceedings of the 37th Annual Meeting of the Cognitive Science Society*; July 22–25, 2015; Pasadena, California, USA.

[ref14] Fugazza C. Moesta A. Pogány Á. Miklósi Á. (2018). Presence and lasting effect of social referencing in dog puppies. Anim. Behav. 141, 67–75. 10.1016/j.anbehav.2018.05.007

[ref15] Gibson J. J. (1977). “Theory of affordances” in Perceiving, acting, and knowing. eds. Shaw R. Bransford J. (London: Routledge), 127–135.

[ref16] Hare B. Tomasello M. (2005). Human-like social skills in dogs? Trends Cogn. Sci. 9, 439–444. 10.1016/j.tics.2005.07.003, PMID: 16061417

[ref17] Hare B. Wobber V. Wrangham R. (2012). The self-domestication hypothesis: evolution of bonobo psychology is due to selection against aggression. Anim. Behav. 83, 573–585. 10.1016/j.anbehav.2011.12.007

[ref18] Hulbert J. C. Anderson M. C. (2008). The role of inhibition in learning. Adv. Psychol. 139, 7–20. 10.1016/S0166-4115(08)10002-4

[ref19] Katayama M. Kubo T. Yamakawa T. Fujiwara K. Nomoto K. Ikeda K.. (2019). Emotional contagion from humans to dogs is facilitated by duration of ownership. Front. Psychol. 10:1678. 10.3389/fpsyg.2019.01678, PMID: 31379690 PMC6658615

[ref20] Kee H. (2020). Pointing the way to social cognition: a phenomenological approach to embodiment, pointing, and imitation in the first year of infancy. J. Theor. Philos. Psychol. 40, 135–154. 10.1037/teo0000130

[ref21] Lakshminarayanan V. R. Santos L. R. (2009). Cognitive preconditions for responses to fairness: an object retrieval test of inhibitory control in capuchin monkeys (*Cebus apella*). J. Neurosci. Psychol. Econ. 2, 12–20. 10.1037/a0015457

[ref22] Macphail E. M. (1970). Serial reversal performance in pigeons: role of inhibition. Learn. Motiv. 1, 401–410. 10.1016/0023-9690(70)90104-9

[ref23] Marshall-Pescini S. Virányi Z. Range F. (2015). The effect of domestication on inhibitory control: wolves and dogs compared. PLoS One 10:e0118469. 10.1371/journal.pone.0118469, PMID: 25714840 PMC4340790

[ref24] McGraw K. O. Wong S. P. (1996). Forming inferences about some Intraclass correlation coefficients. Psychol. Methods 1, 30–46. 10.1037/1082-989X.1.1.30

[ref25] Meltzoff A. N. (2005). “Imitation and other minds: the “like me” hypothesis” in Perspectives on imitation: From neuroscience to social science: Imitation, human development, and culture. Vol. 2 eds. Hurley S. Chater N. (Cambridge: MIT Press), 55–77.

[ref26] Merola I. Prato-Previde E. Lazzaroni M. Marshall-Pescini S. (2014). Dogs’ comprehension of referential emotional expressions: familiar people and familiar emotions are easier. Anim. Cogn. 17, 373–385. 10.1007/s10071-013-0668-1, PMID: 23955027

[ref27] Miklósi Á. (2009). “How to make agents that display believable empathy? An ethological approach to empathic behavior” in *Autonomous Agents and Multi Agent Systems, Proceedings of Workshop 20: Empathic Agents*; May 10–15, 2009; Budapest, Hungary, 43–46.

[ref28] Miklósi Á. Kubinyi E. Topál J. Gácsi M. Virányi Z. Csányi V. (2003). A simple reason for a big difference. Curr. Biol. 13, 763–766. 10.1016/S0960-9822(03)00263-X, PMID: 12725735

[ref29] Miklósi A. Polgárdi R. Topál J. Csányi V. (2000). Intentional behaviour in dog-human communication: an experimental analysis of “showing” behaviour in the dog. Anim. Cogn. 3, 159–166. 10.1007/s100710000072

[ref30] Nagasawa M. Murai K. Mogi K. Kikusui T. (2011). Dogs can discriminate human smiling faces from blank expressions. Anim. Cogn. 14, 525–533. 10.1007/s10071-011-0386-5, PMID: 21359654

[ref31] Nelder J. A. Wedderburn R. W. M. (1972). Generalized linear models. J. R. Stat. Soc. Ser. A 135:370. 10.2307/2344614

[ref32] Petrazzini M. M. E. Mantese F. Prato-Previde E. (2020). Food quantity discrimination in puppies (*Canis lupus familiaris*). Anim. Cogn. 23, 703–710. 10.1007/s10071-020-01378-z 32253517

[ref33] Pongrácz P. Hegedüs D. Sanjurjo B. Kovári A. Miklósi Á. (2013). “We will work for you” - social influence may suppress individual food preferences in a communicative situation in dogs. Learn. Motiv. 44, 270–281. 10.1016/j.lmot.2013.04.004

[ref34] Prato-Previde E. Marshall-Pescini S. Valsecchi P. (2008). Is your choice my choice? The owners’ effect on pet dogs’ (*Canis lupus familiaris*) performance in a food choice task. Anim. Cogn. 11, 167–174. 10.1007/s10071-007-0102-7, PMID: 17641921

[ref35] Range F. Virányi Z. (2014). Wolves are better imitators of conspecifics than dogs. PLoS One 9:e86559. 10.1371/journal.pone.0086559, PMID: 24489744 PMC3906065

[ref36] Repacholi B. M. Gopnik A. (1997). Early reasoning about desires: evidence from 14- and 18-month-olds. Dev. Psychol. 33, 12–21. 10.1037//0012-1649.33.1.12, PMID: 9050386

[ref37] Schmidt M. F. H. Sommerville J. A. (2011). Fairness expectations and altruistic sharing in 15-month-old human infants. PLoS One 6:e23223. 10.1371/journal.pone.0023223, PMID: 22003380 PMC3188955

[ref38] Smit I. Szabo D. Kubinyi E. (2019). Age-related positivity effect on behavioural responses of dogs to human vocalisations. Sci. Rep. 9, 1–10. 10.1038/s41598-019-56636-z, PMID: 31882873 PMC6934484

[ref39] Tauzin T. Csík A. Kis A. Kovács K. Topál J. (2015). The order of ostensive and referential signals affects dogs’ responsiveness when interacting with a human. Anim. Cogn. 18, 975–979. 10.1007/s10071-015-0857-1, PMID: 25771965

[ref40] Topál J. Byrne R. W. Miklósi Á. Csányi V. (2006). Reproducing human actions and action sequences: “do as I do!” in a dog. Anim. Cogn. 9, 355–367. 10.1007/s10071-006-0051-6, PMID: 17024511

[ref41] Topál J. Miklósi Á. Csányi V. Dóka A. (1998). Attachment behavior in dogs (*Canis familiaris*): a new application of Ainsworth’s (1969) strange situation test. J. Comp. Psychol. 112, 219–229. 10.1037/0735-7036.112.3.219, PMID: 9770312

[ref42] Topál J. Miklósi Á. Gácsi M. Dóka A. Pongrácz P. Kubinyi E. (2009). “The dog as a model for understanding human social behavior” in Advances in the study of behavior. eds. Brockmann H. J. Roper T. J. Naguib M. Wynne-Edwards K. E. Mitani J. C. Simmons L. W. (Burlington: Academic Press), 71–116.

[ref43] Turcsán B. Szánthó F. Miklósi Á. Kubinyi E. (2015). Fetching what the owner prefers? Dogs recognize disgust and happiness in human behaviour. Anim. Cogn. 18, 83–94. 10.1007/s10071-014-0779-3, PMID: 24989132

[ref44] Van Bourg J. Patterson J. E. Wynne C. D. L. (2020). Pet dogs (*Canis lupus familiaris*) release their trapped and distressed owners: individual variation and evidence of emotional contagion. PLoS One 15:e0231742. 10.1371/journal.pone.0231742, PMID: 32298391 PMC7162277

[ref45] Warneken F. (2006). Altruistic helping in human infants and young chimpanzees. Science 311, 1301–1303. 10.1126/science.1121448, PMID: 16513986

[ref46] Warneken F. Tomasello M. (2009). Varieties of altruism in children and chimpanzees. Trends Cogn. Sci. 13, 397–402. 10.1016/j.tics.2009.06.008 19716750

[ref47] Wynn K. Bloom P. Jordan A. Marshall J. Sheskin M. (2018). Not noble savages after all: limits to early altruism. Curr. Dir. Psychol. Sci. 27, 3–8. 10.1177/0963721417734875 29713124 PMC5921922

